# *In vitro* effectiveness of pomegranate extract present in pet oral hygiene products against canine oral bacterial species

**DOI:** 10.14202/vetworld.2022.1714-1718

**Published:** 2022-07-20

**Authors:** Abish S. Stephen, Celine S. Nicolas, Fanny Lloret, Robert P. Allaker

**Affiliations:** 1 Centre for Oral Immunobiology and Regenerative Medicine, Institute of Dentistry, Queen Mary University of London, London, UK; 2 Global Marketing and Market Development, Virbac, Carros, France; 3 Petcare Products Development Unit, Virbac, Carros, France

**Keywords:** dental biofilm, dogs, *Neisseria canis*, oral hygiene, pomegranate, *Porphyromonas gulae*

## Abstract

**Background and Aim:**

Pomegranate is known to possess antibacterial properties, partly because of its punicalagin content. However, its effect on canine oral bacterial species has not yet been elucidated. In this study, we evaluated the effect of pomegranate extract present in pet dental products on the growth and survival of five canine oral bacterial species in biofilms.

**Materials and Methods:**

Five bacterial species, *Neisseria shayeganii*, *Neisseria canis*, *Porphyromonas gulae*, *Porphyromonas macacae*, and *Porphyromonas crevioricanis*, were individually cultured for biofilm formation and exposed to pomegranate extract (or control) for 15 min. Cell survival was analyzed using the 3-(4,5-dimethylthiazol-2-yl)-2,5-diphenyltetrazolium bromide assay and was compared between different conditions using a student’s t-test. In addition, the individual strains were grown in planktonic suspensions and exposed to serial dilutions of the extract to determine the minimum inhibitory concentration.

**Results:**

At a concentration of 0.035% w/v, the extract significantly reduced the survival of *P. gulae* (−39%, p < 0.001) and *N. canis* (−28%, p = 0.08) in biofilms. At similar concentrations, the extract also completely or partially inhibited the growth of *N. canis and Porphyromonas* spp. in planktonic suspensions, respectively.

**Conclusion:**

The pomegranate extract found in some pet dental products can limit bacterial growth and survival in the biofilms formed by *N. canis* and *P. gulae in vitro*. As *P. gulae* is involved in periodontal disease progression, limiting its proliferation using products containing pomegranate extract could contribute to disease prevention. Further studies on dogs receiving such products are necessary to confirm these effects.

## Introduction

Periodontal disease (PD) can include gingivitis, which is the initial and reversible stage of inflammation of the gingiva, and periodontitis, which is the later stage of the inflammatory disease that involves the deeper structures of the tooth, resulting in the progressive destruction of the periodontal tissue and subsequent loss of attachment [1]. PD is common in dogs and cats and has deleterious effects, including pain and tooth loss; moreover, it can lead to serious local and systemic consequences [1, 2]. The growth of oral bacteria forming biofilms, which thickens into a plaque and leads to tartar formation on the dental enamel, is known to contribute to periodontitis. Dental plaque developing in the gingiva can stimulate the immune system and lead to gingivitis, which is the first, reversible stage of PD. If left untreated, the condition can progress into periodontitis, which results in the destruction of periodontal tissues [1, 2]. Although the removal of plaque by mechanical action (tooth brushing) is the best prevention approach, the prevention of bacterial adherence can also control plaque formation, particularly in species or individuals who have difficulties with tooth brushing.

Pomegranate (*Punica granatum*) is known to possess antioxidant properties as it contains polyphenols, including flavonoids (anthocyanins), and tannins, such as punicalagin and ellagic acid [3–5]. Tannins, such as punicalagin, are known to exhibit antimicrobial effects [5–7]. Pomegranate extracts have been shown to exhibit beneficial effects on oral bacterial strains in humans [5, 8–12]. However, their effect against canine oral bacterial strains involved in PD has not yet been evaluated. In this study, the effects of the extract present in the water additive Aquadent^®^ FR3SH™ (Virbac, France) and the dental chew Veggiedent^®^ FR3SH™ (Virbac) on the growth and plaque formation of canine oral bacterial strains were tested *in vitro*. Two strains of *Neisseria* and three strains of *Porphyromonas* were evaluated. The *Neisseria* spp. (aerobes) are early plaque colonizers that can help the binding of late colonizers which are associated with periodontitis; *Porphyromonas* spp. (anaerobes), which are often found in subgingival plaque, are associated with PD progression [13–16].

This study aimed to determine for the first time whether pomegranate extract can limit the *in vitro* growth and survival in biofilms of canine oral bacterial strains involved in PD in dogs at concentrations found in pet dental products to inform its ability to be used for PD prevention.

## Materials and Methods

### Ethical approval

This is an *in vitro* study; the study did not involve any human or animal subject, so ethical approval was not necessary.

### Study period and location

This study was performed from November 2019 to February 2021 at the Centre for Oral Immunobiology and Regenerative Medicine, Institute of Dentistry, Queen Mary University of London, London, UK.

### Organisms and culture conditions (*in vitro*)

The five strains, *Neisseria shayeganii* (DSM 22246), *Neisseria canis* (DSM 18000*), Porphyromonas gulae* (DSM 15663)*, Porphyromonas macacae* (DSM 20710), and *Porphyromonas crevioricanis* (DSM 104771), were supplied by the Glasgow Dental School (UK).

*Neisseria* spp. and *Porphyromonas* spp. bacteria were maintained on blood agar (with 5% v/v defibrinated horse blood; Oxoid, UK) at 37°C in aerobic (5% CO_2_) and anaerobic (85% N_2_; 10% CO_2_; 5% H_2_) conditions, respectively. Liquid cultures used to prepare bacterial suspensions for antibiofilm or minimum inhibitory concentration (MIC) assays were grown overnight on Brain Heart Infusion (BHI, Oxoid) supplemented with Hemin (0.0005% w/v; Sigma, UK).

### *In vitro* biofilm assay

Bacterial cultures incubated aerobically or anaerobically at 37°C for 1 or 2 days, respectively, were pelleted by centrifugation (3,000× *g* for 10 min at 20°C). The pellets were resuspended in BHI Hemin broth and standardized at a concentration of 10^7^ colony-forming units per mL at an optical density (OD) of 600 nm (OD_600_). Biofilms were formed in microtiter plate wells (Corning, UK) by incubating 100 μL of standardized inoculum for 90 min at 37°C under shaking conditions (75 rpm) to obtain pre-adhesion [17]. The supernatant was then discarded and 200 mL of broth was added for biofilm growth. The broth was replaced every 24 h during the incubation at 37°C. The incubation period was 24 h and 48 h for aerobes and anaerobes, respectively.

The biofilms were then exposed to 100 μL of pomegranate peel extract at 0.035% w/v which is equivalent to the concentration found in Veggiedent FR3SH chews and Aquadent FR3SH water additive formulas (Virbac) – or the negative control (deionized water) for 15 min. The pomegranate extract was obtained through hydroalcoholic extraction from *P. granatum* fruit skin, which was then purified, concentrated, and spray-dried to obtain free-flowing powder. The percentage of surviving cells after exposure was estimated by the analysis of the 3-(4,5-dimethylthiazol-2-yl)-2,5-diphenyltetrazolium bromide (MTT) assay (Abcam, UK; ab211091), according to the manufacturer’s instructions. The test solutions were removed by pipetting from the wells and then 50 μL of MTT solution and 50 μL of medium were added to the wells, and the plate was incubated aerobically or anaerobically at 37°C for 1 h under light protection. After incubation, 150 μL of MTT solvent solution was added for the solubilization of products formed due to the biochemical activity of the biofilm viable cells. After 15 min of incubation at 37°C under shaking conditions in an orbital shaker (approximately 75 rpm), the OD was obtained (λ = 590 nm) using a microplate spectrophotometer (BMG Labtech CLARIOStar Plus, Ortenberg, Germany).

Experiments were repeated three times, and the treatments were performed in quadruplicate biofilms per experiment and per strain to obtain a total of 12 OD readings per treatment and per strain.

### Assessment of MIC *in vitro*

The effect of the pomegranate extract on bacterial growth was determined using a broth microdilution assay [18]. Bacterial suspensions at an OD_600_ of 0.1 and 0.2 for the aerobic and anaerobic strains, respectively, were prepared from pelleted broth cultures (3000× *g* for 10 min at 20°C). Serial 2-fold dilutions of the pomegranate extract were prepared in a total broth volume of 90 μL per well of the microtiter plate (8 concentrations tested in total). The bacterial suspension (90 μL) and medium (90 μL) were then added to each well before incubation at 37°C for 24 h. The OD at 600 nm was read before and after the incubation using a microplate spectrophotometer (BMG Labtech CLARIOStar Plus). Experiments were repeated three times for the aerobes (n = 3 per concentration tested) and four times for the anaerobes (n = 4 per concentration tested).

### Statistical analysis

For the biofilm assay, the OD values were normalized within each plate and compared between different conditions using student’s t-test. The normality of data distribution was assessed using D’agostino-Pearson’s test. The significance level was set at 5% (α = 0.05). The analysis was performed using GraphPad Prism (v9.1.0; GraphPad Software, San Diego, USA).

## Results

### Biofilm assay

The biofilms formed by each strain were exposed to the pomegranate extract or negative control for 15 min. The amount of cells surviving after each exposure was then measured and compared between the treatments.

**Figure-1:**
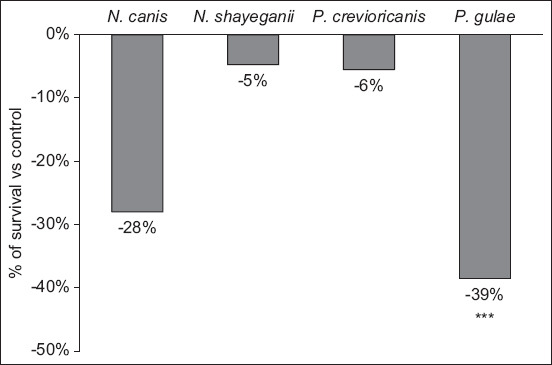
Survival of cells in biofilms after exposure to the pomegranate extract. The data are expressed as the percentage of survival for each strain, based on the reduction in mean optical density (OD) values in the treated biofilms versus control. ***p < 0.001: Statistically significant difference in OD values versus control (student’s t-test).

Among the aerobic strains, compared with the control, the survival of *N. canis* decreased by 28% when exposed to pomegranate extract (p = 0.08), and the survival of *N. shayeganii* was not impacted by the extract (−5%; Figure-1). Among the anaerobic strains tested, compared with the control, the survival of *P. gulae* significantly decreased by 39% when exposed to pomegranate extract (p < 0.001), and the survival of *P. crevioricanis* was not impacted by the extract (−6%; Figure-1).

### Bacterial growth inhibition

**Figure-2:**
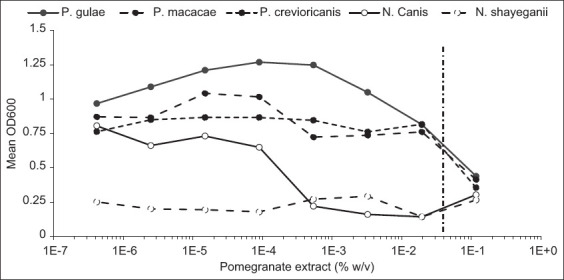
Growth inhibition of the bacterial strains tested by different concentrations of the pomegranate extract. Data are expressed as the mean optical density at the different concentrations of the extract tested. The vertical dotted line represents the concentration of the extract equivalent to what is found in Aquadent FR3SH and Veggiedent FR3SH.

The bacterial strains in suspension were exposed to serial dilutions of the pomegranate extract. At the highest concentration (0.116% w/v), the growth of all strains (except *N. shayeganii*, for which the data were inconclusive) was partially or completely inhibited (Figure-2). At the concentration found in Veggiedent FR3SH chews and Aquadent FR3SH solution (0.035% w/v, vertical dotted line in Figure-2), the growth of *N. canis* remained completely inhibited and the growth of the other strains was partially inhibited (Figure-2). Based on the inhibition curves, the MIC of the pomegranate extract for *N. canis* was determined to be 0.00048% (w/v). The non-inhibitory concentrations of pomegranate extract were 0.00009% and 0.0061% for *N. canis* and *P. gulae*, respectively.

## Discussion

The effect of pomegranate extract on bacterial growth in plaque or suspension was tested *in vitro* for bacterial strains commonly found in the canine oral cavity. At the concentration of the extract found in Veggiedent FR3SH and Aquadent FR3SH (0.035%), a significant decrease was observed in the survival of the pathogenic strain *P. gulae* compared with the control. Although the MIC could not be determined, partial inhibition of bacterial growth was observed when the bacteria were cultured with the extract at similar concentrations. The survival of *N. canis* was also impacted by the pomegranate extract, although the decrease in survival was not statistically significant compared with the control (p = 0.08). However, the MIC of the extract on this strain was approximately 0.00048% (w/v) and complete growth inhibition was observed at the concentration of the extract used in canine oral hygiene products. At this concentration, the partial growth inhibition of *P. crevioricanis* and *P. macacae* may also be expected (Figure-2). These data suggest that the pomegranate extract can help limit bacterial growth and cell survival in *N. canis* and *P. gulae* biofilms.

Similar antibacterial effects of pomegranate have been observed against human oral bacterial strains with different sensitivities [5, 8–12]. In some previous studies, the effects of pomegranate extracts were tested against *Porphyromonas gingivalis*, a species related to *P. gulae*, either *in vitro* [11, 19, 20] or in animal models [21]. One of these studies reported an MIC of 170 mg/mL (0.017% w/v) [20], whereas the other study reported an MIC of 31.25 mg/mL (3.125% w/v) [11]. Such a discrepancy could be explained by the possible difference in the extract used in the studies. Indeed, the intensity of the antibacterial effects depends on the polyphenol content, which can vary depending on the fruit part that is extracted and the extraction method used [6, 7]. Peel extracts, for example, have the highest phenolic content and the highest antimicrobial and antioxidant activities [7]. In addition, these extracts are complex mixtures of compounds, including punicosides, punicalagins, and polyphenols, which may act synergistically to exhibit a broader range of antimicrobial activities against Gram-positive and Gram-negative bacteria [22]. The exact mechanism by which pomegranate exerts its antimicrobial effects remains unclear; however, it may involve membrane disruption or interactions with metal ions and proteins such as enzymes [22]. Further studies are needed to better understand the mode of action of each active component and the combination of the active components.

To the best of our knowledge, the antibacterial effects of the pomegranate extract on oral bacterial strains of dogs have not yet been elucidated. Such an effect also needs to be confirmed *in vivo*. Indeed, limiting the bacterial growth of *N. canis* and *P. gulae* could help limit plaque development and PD as these strains, particularly *P. gulae*, have been shown to play a role in microbial dysbiosis associated with PD progression [13–16]. The antioxidant effects of pomegranate on oral bacterial strains are of particular interest for oral hygiene as PD results from an imbalance between bacterial homeostasis and the inflammatory response by the immune system [2, 23].

## Conclusion

In conclusion, this is the first study to assess the effect of pomegranate extract against canine oral bacterial strains *in vitro*. This study suggests that pet dental products containing this extract could be used for PD prevention. Evaluating the effects of such products on bacterial proliferation in the mouths of canines, plaque and tartar accumulation, and PD prevention is needed to further support this finding.

## Authors’ Contributions

ASS, CSN, FL, and RPA: Planned and designed the study, provided resources, and revised the manuscript. ASS and RPA: Realized the study. ASS, CSN, and RPA: Analyzed the data and prepared the manuscript. RPA and CSN: Supervised the study. All authors have read and approved the final manuscript.
